# Interprofessional Collaboration Between Nurses and Occupational Therapists Enhances Independence and Reduces Disposable Absorbent Product Use in Older Patients

**DOI:** 10.1155/oti/4450215

**Published:** 2026-01-04

**Authors:** Ken Kondo, Naoto Noguchi, Ryoto Akiyama, Takahiro Otsuka, Chidawan Suyakong, Shunya Honda, Naomi Tajima, Waka Murata, Bumsuk Lee

**Affiliations:** ^1^ Department of Occupational Therapy Faculty of Rehabilitation, Gunma Paz University, Takasaki, Gunma, Japan, paz.ac.jp; ^2^ Graduate School of Health Sciences, Gunma University, Maebashi, Gunma, Japan, gunma-u.ac.jp; ^3^ Department of Rehabilitation, Gunma Rehabilitation Hospital, Nakanojo, Gunma, Japan; ^4^ Graduate School of Health Sciences Doctor’s Program, Gunma University, Maebashi, Gunma, Japan, gunma-u.ac.jp; ^5^ Department of Rehabilitation, Gunma Paz Hospital, Tone, Gunma, Japan

**Keywords:** activities of daily living, collaborative practice, disposable absorbent products, nurse, occupational therapist

## Abstract

**Introduction:**

The prospective, historically controlled study evaluated whether a collaborative practice (CP) model between nurses and occupational therapists improves activities of daily living (ADLs) and reduces the use of disposable absorbent products and physical restraints in hospitalized older patients.

**Methods:**

Data from the historical control group (*n* = 72), who received usual care, were collected from medical records, and the intervention group (*n* = 46), who participated in the CP‐based intervention, was recruited in a community‐based care ward in a regional hospital. The CP model was designed to facilitate collaborative planning for improving ADLs between nurses and occupational therapists. Outcome measures included disposable absorbent product use, physical restraint use, and the functional independence measure (FIM). Assessments were conducted at admission and discharge. Propensity score matching was applied to balance baseline characteristics between groups and to reduce potential confounding factors.

**Results:**

Propensity score matching generated 45 pairs (“historical controls,” *n* = 45, and “interventions,” *n* = 45). Although physical restraint use was reduced in both groups (*p* ≤ 0.007), the use of disposable absorbent products in the intervention group was significantly reduced compared to the historical control group (*p* = 0.020). Additionally, significant interaction effects were observed between time and group for all FIM scores, indicating greater improvements in ADLs in the intervention group, with moderate to large effect sizes (*p* ≤ 0.013, partial *η* ^2^ ≥ 0.068).

**Conclusions:**

This study demonstrated the positive impact of a CP model between nurses and occupational therapists in improving ADLs and reducing disposable absorbent product use in older patients. These findings suggest that this model of CP enhances the quality of geriatric care.

**Trial Registration:** UMIN Clinical Trials Registry number: UMIN000047072.

## 1. Introduction

Older people are vulnerable to activity restrictions in hospital settings, which causes negative effects [1, 2]. For example, a reduction in activity during hospital stays often leads to passivity and immobility [3, 4]. As a result, older patients often experience a loss of activities of daily living (ADLs) compared to their pre‐admission levels [5].

Both physical restraints and disposable absorbent products are typical measures to restrict patient movement [6, 7], but through different mechanisms. Whereas physical restraints directly limit voluntary movement, primarily to prevent falls or interference with medical devices [8], absorbent products, especially adult briefs with tape tabs, indirectly restrict mobility by limiting access to the toilet and promoting prolonged bed rest. Although healthcare professionals perceive them as less restrictive than physical restraints [9], they negatively impact physical, psychological, and emotional well‐being. For example, patients who wear adult briefs with tape tabs tend to remain in prolonged bed immobility, even if they retain the ability to use the toilet [10]. Additionally, invading personal space when changing this type of absorbent product can cause embarrassment, discomfort, and loss of dignity [11, 12].

Given these adverse effects, prolonged absorbent product use may delay rehabilitation and hinder ADL recovery [7]. However, discontinuing absorbent product use in hospitals is a challenge [13]. One reason is that patients with limited mobility often accept absorbent product use due to concerns about soiling their clothes or the risk of falling, particularly during nighttime [13]. Nursing staff also face challenges in providing toileting assistance. While continence care is required in a timely fashion, predicting the exact timing of toileting needs is difficult [9, 14]. Moreover, nursing staff tend to hesitate to assist with toileting because of limited assistance skills compared to rehabilitation staff [15]. Thus, addressing these complex challenges requires collaboration with rehabilitation staff, as nursing staff alone may struggle to reduce absorbent product use.

Occupational therapists can support nursing staff in managing patients’ self‐care in hospital settings. Occupational therapy interventions play a crucial role in improving patients’ ADLs in rehabilitation settings [16]. Furthermore, occupational therapists emphasize a holistic approach to help patients recover their independence in their daily lives [17]. In other words, occupational therapy techniques can help nurses deliver comprehensive patient care. Several studies have highlighted the importance of nurse‐occupational therapist collaboration [15, 18]. In a previous pilot study, we introduced a collaborative practice (CP) model between nurses and occupational therapists to improve older patients’ ADLs as well as their social cognitive function and motivation related to ADLs [19]. However, whether this type of CP enhances older patients’ independence and reduces reliance on absorbent products and physical restraints remains unexplored.

This study aimed to evaluate the impact of a CP model between nurses and occupational therapists for the recovery of ADLs, as well as its role in reducing the use of disposable absorbent products and physical restraints. Based on our previous research [19], the study was conducted in a community‐based care ward to assess its effectiveness. Although rehabilitation services are mainly provided in convalescent rehabilitation wards in Japan, these wards more focus their services on functional recovery rather than ADL recovery. On the other hand, the community‐based care wards relatively prioritize ADL recovery itself to facilitate early discharge, because the wards mainly accept elderly patients who want to return home even without functional recovery [20]. Since both our previous and present studies focused on ADL recovery among older patients, we considered the community‐based care ward to be the optimal setting in which to examine the effectiveness of the CP model. By examining the CP in this context, our study contributes to evidence‐based strategies for optimizing geriatric care.

## 2. Methods

### 2.1. Study Design, Setting, and Participants

This study is a prospective, historically controlled study. The study was approved by the institutional review boards of the university (PAZ21‐36) and the hospital (No.33). This study is reported in accordance with the Transparent Reporting of Evaluations with Nonrandomized Designs (TREND) statement, which provides guidelines to ensure transparency and quality in reporting nonrandomized intervention studies [21].

Participants in the intervention were recruited from a community‐based care ward in a hospital between June 1, 2022, and June 30, 2023. Historical controls were admitted to the ward before the initiation of the intervention, and their data were collected from medical records spanning the period between April 1, 2020, and March 31, 2022. The community‐based care ward is a new type of hospital unit launched in Japan in 2014 to provide older patients with intensive rehabilitation services in the subacute phase. Inclusion criteria were patients who received rehabilitation therapy and were aged 65 years or older. Exclusion criteria included terminal cancer, lack of informed consent, transfer to other wards, death during hospital stay, and insufficient clinical assessments. The study coordinator identified eligible patients from the hospital database and contacted them directly to explain the study or, if necessary, reached out to their family members. Eligible patients were given sufficient time to decide on participation. Informed consent was obtained before the intervention.

### 2.2. Interventions

Based on our previous study [19], we developed the CP model to foster interprofessional collaboration between nurses and occupational therapists. In the intervention group, only patients who were hospitalized at the time were discussed during the weekly multidisciplinary team (MDT) meetings. At these meetings, their clinical progress and care plans were reviewed without patient participation. Subsequently, these patients underwent further evaluation through the CP model. Notably, this CP‐based intervention was implemented only once during the hospital stay and consisted of two phases: (1) a nurse–occupational therapist meeting, conducted without patient participation, and (2) a hands‐on demonstration, which involved patient participation.

In the first phase, the nurse‐occupational therapist meeting, separate from the weekly MDT meetings, was conducted in accordance with the situation–background–assessment–recommendation (SBAR) technique [22]. This framework was chosen to ensure structured and effective communication between nurses and occupational therapists, facilitating co‐planning for improving the patient’s ADL. Before the meeting, occupational therapists assessed a patient’s maximum abilities, preferences, and needs and pre‐hospital meaningful occupations (e.g., hobbies, work, social roles). During the meeting, nurses and occupational therapists began by sharing the patient’s clinical and social characteristics (situation and background). Next, they collaboratively assessed the patient’s motor and social‐cognitive functions using the functional independence measure (FIM). In addition, each profession contributed clinical assessments based on their respective expertise (assessment). Once a mutual understanding of the patient’s current state was achieved, co‐planning for the improvement of the patient’s ADL was discussed (recommendation). Approximately 15 min were allocated per patient.

In the second phase, detailed feedback was provided to the other nurses and the responsible therapists. To ensure the patient’s understanding and agreement with the plan, an explanation of the scope of practice was given, followed by the hands‐on demonstration involving the patient, nurses, and occupational therapists in the patient’s room.

### 2.3. Historical Controls

The historical controls were selected using inclusion and exclusion criteria similar to those applied to the intervention group. They received usual hospital care, including rehabilitation therapy, comprehensive nursing care, and a weekly MDT meeting, and did not receive the CP‐based intervention. In the weekly MDT meeting, assessment data related to each patient’s medical condition were shared among healthcare professionals. Nurses reported patients’ current health issues, a social worker reported the socioeconomic status, and a representative therapist reported the functional capability, which was documented by responsible therapists. Based on their reports, a physician decided on a final treatment policy. Approximately 5 min were allocated for each patient. The opt‐out notice was posted on the hospital website for historical control patients, and the requirement to obtain informed consent was waived.

### 2.4. Outcomes

Socio‐demographic and clinical characteristics for the study included age, sex, living state (in their own home or a facility), family living together in home‐living patients (alone or with family), the length of stay in the acute ward, admission diagnosis, diagnosis of dementia, and the Charlson comorbidity index [23]. In addition, the amount of rehabilitation therapy, the length of stay in the community‐based care ward, and the discharge destination were included.

Disposable absorbent products and physical restraints were monitored from nursing records at admission and discharge. In this study, we focused specifically on adult briefs with tape tabs, as they pose greater barriers to toilet use compared to pull‐up briefs or incontinence pads. In addition, as our interest was focused on physical restraint to limit voluntary movement, any devices to restrict patient movement, including both bed‐related and wheelchair restraints, were regarded as physical restraints in this study.

Each patient’s ADL and social‐cognitive functions were assessed using the FIM [24]. It consists of 13 motor and five cognitive items. The motor section has four subscales: Self‐care, Sphincter, Transfer, and Locomotion. The cognitive section has two subscales: Communication and Social cognition. The FIM score ranges from 1 (dependence) to 7 (independence) for 18 items with a maximum score of 126 that indicates total functional independence. The reliability and validity of the FIM have been demonstrated [24]. Two FIM‐related parameters were also used: the FIM gain and FIM efficiency [25]. The FIM gain was defined as each score at discharge minus each score at admission. This reflects functional improvement during hospitalization. The FIM efficiency was defined as each gain divided by the length of the hospital stay. This reflects the rate of functional improvement. The assessments were delivered within 72 h after admission and no more than 72 h before discharge, and the evaluators were occupational therapists who were familiar with using the FIM.

### 2.5. Sample Size

The calculation of statistical power was performed a priori using G∗power 3.1.9.2. The study was conducted to determine the median effect with Cohen’s *f* = 0.25 and to ensure a sufficient power of 0.80. Therefore, the sample size calculation indicated 49 historical control patients and 49 intervention patients. To account for a potential dropout of 20%–30% [26], 68 patients were enrolled in the intervention group. In the historical control group, we collected data with a sample size similar to that of the intervention group.

### 2.6. Statistical Methods

We performed propensity score matching to reduce the potential for confounding factors [27]. Propensity scores were estimated using logistic regression analysis, and 1:1 patient matching with a caliper of 0.2 standard deviations was applied. The variables included in the propensity score analysis were age, sex, the use of disposable absorbent products and physical restraints at admission, and the initial FIM motor and cognitive subscale scores as these covariates were assumed to have a potential confounding influence on the outcomes.

After adjusting for confounding factors through propensity score matching, the chi‐square test or the unpaired *t* test was used to compare the two groups’ socio‐demographic and clinical characteristics. For the analysis of the use of disposable absorbent products and physical restraints, within‐group analyses were performed using the McNemar test between different time points, and the chi‐square test was used to compare the two groups.

The split‐plot ANOVA and the chi‐square test were used to compare the two groups at two time points. Effect size estimates were reported as partial eta squared (partial *η* ^2^). The thresholds for partial *η* ^2^ were as follows: 0.01–0.06 for small, 0.06–0.14 for moderate, and > 0.14 for large [27].

The unpaired *t* test was used to compare the FIM gain and efficiency scores between the two groups. The effect size (Cohen’s *d*) was calculated for each outcome variable to indicate the magnitude of a performance difference. The thresholds for Cohen’s *d* were as follows: < 0.20 for trivial, 0.21–0.50 for small, 0.51–0.80 for moderate, and > 0.81 for large [28].

Statistical analyses were performed using IBM SPSS Statistics for Windows, version 29. Significance was set at *p* < 0.05.

## 3. Results

**Figure 1:**
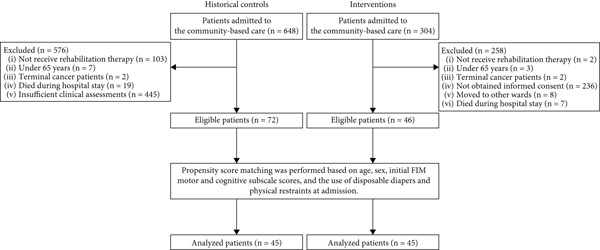
Participant flow throughout the study. Alt text: Flowchart of the process for participant assignment.

Figure 1 shows the flow diagram of the process of study selection. In the historical control group, of the 648 patients admitted to the community‐based care ward, 576 patients did not meet the inclusion criteria: patients who did not receive rehabilitation therapy owing to restrictions during the earlier period of the pandemic (*n* = 103), patients who aged under 65 years (*n* = 7), terminal cancer patients (*n* = 2), patients who died during hospital stay (*n* = 19), and patients with insufficient clinical assessments (*n* = 445). The remaining 72 patients were analyzed.

In the intervention group, of the 304 patients admitted to the community‐based care ward, 258 patients did not meet the inclusion criteria. The reasons for exclusion were as follows: patients who did not receive rehabilitation therapy (*n* = 2), patients who aged under 65 years (*n* = 3), terminal cancer patients (*n* = 2) and patients who did not give informed consent (*n* = 236), patients who transferred to other wards (*n* = 8) and patients who died during their hospital stay (*n* = 7) were excluded. The remaining 46 patients were analyzed. No adverse events occurred during the intervention, supporting its safety. Overall, 45 pairs were selected from the historical control and intervention groups through propensity score matching.

Baseline socio‐demographic and clinical characteristics are summarized in Table 1. The mean ages of the historical control and intervention groups were 88.2 ± 7.2 years with 12 males and 33 females, and 89.2 ± 6.6 years with 12 males and 33 females, respectively. The baseline characteristics between the two groups did not differ significantly.

**Table 1:** Socio‐demographic and clinical characteristics of the participants.

	**Historical controls (** **n** = 45**)**	**Interventions (** **n** = 45**)**	***p***
Age, year, mean (SD)	88.2 (7.2)	89.2 (6.6)	0.518
Sex, number, male/female	12/33	12/33	1.000
Living state, number, home/facility	7/38	13/32	0.128
Family living together in home‐living patients, number, alone/with family	2/5	5/8	0.350
Length of stay in the acute ward, day, mean (SD)	8.2 (12.0)	8.8 (5.5)	0.752
Admission diagnosis, number (%)			
Cardiopulmonary disorder	23 (51.1)	16 (35.6)	0.136
Musculoskeletal disorder	4 (8.9)	9 (20.0)	0.134
Infection disease	5 (11.1)	3 (6.7)	0.459
Neurological disease	3 (6.7)	1 (2.2)	0.306
Other	10 (22.2)	16 (35.6)	0.163
Dementia, number (%)	14 (31.1)	17 (37.8)	0.506
Charlson comorbidity index, mean (SD)	1.9 (1.7)	1.8 (1.6)	0.800
Amount of rehabilitation therapy received, min, mean (SD)	1432.4 (1120.7)	1819.1 (1100.7)	0.102
Length of stay, days in the community‐based care ward, mean (SD)	26.3 (17.8)	41.8 (19.2)	**< 0.001**
Discharge destination, number, home/others	5/40	11/34	0.098
Number of weekly meetings, mean (SD)	3.6 (2.5)	6.0 (2.7)	**< 0.001**
Number of days from admission to nurse‐occupational therapist meeting, mean (SD)	—	25.3 (16.8)	

*Note:* Values in bold indicate a significant difference.

The length of stay in the community‐based care ward in the intervention group was longer than in the historical control group. A significant difference was not detected in the amount of rehabilitation therapy and the discharge destination between the two groups. During the hospital stay, the historical controls were reviewed in a weekly MDT meeting an average of 3.6 ± 2.5 times. The patients in the intervention group were reviewed in the MDT meeting an average of 6.0 ± 2.7 times. The nurse‐occupational therapist meeting was held 25.3±16.8 days after admission.

Table 2 shows the results of comparisons within and between the two groups in the rate of disposable absorbent product use and physical restraint use. The rate of disposable absorbent product use showed a significant reduction only in the intervention group (*p* = 0.008). In addition, the rate of disposable absorbent product use in the intervention group was lower than that of the historical control group at discharge (*p* = 0.020). On the other hand, whereas both groups showed a significant reduction in the rate of physical restraint use (*p* ≤ 0.007), the use of physical restraints at discharge between the two groups did not differ significantly. The primary purpose of physical restraint in both groups was fall prevention (e.g., bed exit alarm, full enclosure bed, rails, wheelchair belt) at admission. At discharge, the same tendencies observed at admission were seen in both groups.

**Table 2:** Summary of changes in disposable absorbent product use and physical restraint use at admission and discharge.

	**Historical controls (** **n** = 45**)**			**Interventions (** **n** = 45**)**			**Historical controls vs. interventions**	
	**Admission**	**Discharge**		**Admission**	**Discharge**		**Admission**	**Discharge**
			***p***			***p***	***p***	***p***
Disposable absorbent product use, number (%)	33 (73.3)	30 (66.7)	0.375	27 (60.0)	19 (42.2)	**0.008**	0.180	**0.020**
Physical restraint use, number (%)	22 (48.9)	12 (26.7)	**0.006**	21 (46.7)	10 (22.2)	**0.007**	0.833	0.624

*Note:* Values in bold indicate a significant difference.

Table 3 presents the results of comparisons within and between the two groups. The split‐plot ANOVA revealed a significant main effect of time in all FIM‐related scores with moderate to large effect sizes (*p* < 0.001, partial *η* ^2^ ≥ 0.118), indicating improvement in these scores in both groups. The significant main effect of the group was found in the FIM total, the FIM motor Sphincter, the FIM motor Transfer, and the FIM cognitive Social cognition with small to moderate effect sizes (*p* ≤ 0.047, partial *η* ^2^ ≥ 0.044), indicating additional improvement in these scores in the intervention group. Significant interaction effects between time and group were observed in all FIM scores with moderate to large effect sizes (*p* ≤ 0.013, partial *η* ^2^ ≥ 0.068). Table 4 shows the results of comparisons between the two groups in the FIM gain and efficiency. The intervention group gained additional improvements in the FIM gain total, motor, and cognitive scores with moderate to large effect sizes (*p* < 0.001, Cohen^’^s *d* ≥ 0.79), and the FIM efficiency in cognitive score with a moderate effect size (*p* = 0.010, Cohen^’^s *d* = 0.57).

**Table 3:** Summary of outcome measurements at admission and discharge.

	**Historical controls (** **n** = 45**)**		**Interventions (** **n** = 45**)**		**Time effect**			**Group effect**			**Interaction**		
	**Admission**	**Discharge**	**Admission**	**Discharge**	***F***	***p***	**Partial *η*** ^**2**^	***F***	***p***	**Partial *η*** ^**2**^	***F***	***P***	**Partial *η*** ^**2**^
FIM, mean (SD)													
Total	45.6 (22.6)	48.2 (24.6)	48.7 (24.6)	64.6 (25.8)	54.429	**< 0.001**	0.382	4.062	**0.047**	0.044	28.118	**< 0.001**	0.242
Motor	28.2 (16.8)	30.6 (18.8)	29.8 (16.2)	43.3 (20.1)	49.310	**< 0.001**	0.359	3.747	0.056	0.041	24.075	**< 0.001**	0.215
Self‐care	14.7 (7.8)	15.8 (8.5)	14.8 (7.6)	20.9 (8.9)	59.947	**< 0.001**	0.384	2.425	0.123	0.027	26.996	**< 0.001**	0.235
Sphincter	4.3 (3.8)	4.6 (4.1)	5.6 (4.4)	7.4 (5.0)	12.435	**< 0.001**	0.124	5.634	**0.020**	0.060	6.399	**0.013**	0.068
Transfer	6.2 (4.3)	7.1 (5.1)	6.8 (4.1)	10.3 (5.3)	39.102	**< 0.001**	0.308	4.286	**0.041**	0.046	14.466	**< 0.001**	0.141
Locomotion	3.0 (1.8)	3.2 (1.9)	2.6 (1.3)	4.8 (3.2)	25.973	**< 0.001**	0.228	2.053	0.155	0.023	18.168	**< 0.001**	0.171
Cognitive	17.4 (7.0)	17.6 (6.8)	18.9 (6.4)	21.1 (6.9)	19.848	**< 0.001**	0.184	3.328	0.072	0.036	14.121	**< 0.001**	0.138
Communication	8.6 (3.3)	8.6 (3.2)	8.5 (2.1)	9.3 (2.6)	11.803	**< 0.001**	0.118	0.351	0.555	0.004	8.451	**0.005**	0.088
Social cognition	8.9 (4.0)	9.0 (4.0)	10.4 (4.6)	11.9 (4.7)	14.544	**< 0.001**	0.142	6.129	**0.015**	0.065	10.314	**0.002**	0.105

*Note:* Values in bold indicate a significant difference.

Abbreviation: FIM, functional independence measure.

**Table 4:** Summary of comparisons between the two groups in the FIM gain and efficiency.

	**Historical controls (** **n** = 45**)**	**Interventions (** **n** = 45**)**	**Mean difference (95% CI)**	***p***	**Cohen’s *d***
FIM gain, mean (SD)					
Total	2.6 (8.2)	15.9 (14.7)	13.3 (8.309–18.269)	**< 0.001**	1.12
Motor	2.4 (7.9)	13.5 (13.0)	11.1 (6.624–15.643)	**< 0.001**	1.03
Cognitive	0.2 (1.1)	2.4 (3.7)	2.2 (1.016–3.296)	**< 0.001**	0.79
FIM efficiency, mean (SD)					
Total	0.6 (3.6)	0.5 (0.6)	0.0 (− 1.108–1.040)	0.950	0.01
Motor	0.6 (3.6)	0.5 (0.5)	− 0.1 (− 1.177–0.963)	0.843	0.04
Cognitive	0.0 (0.0)	0.1 (0.2)	0.1 (0.182–0.128)	**0.010**	0.57

*Note:* Values in bold indicate a significant difference.

Abbreviation: FIM, functional independence measure.

## 4. Discussion

We found that the intervention group used as disposable absorbent products at discharge at a lower rate than the historical control group. Moreover, the FIM gain total, including both motor and cognitive components, and the FIM cognitive efficiency was significantly higher in the intervention group than in the historical control group.

Disposable absorbent products are widely used continence aids in hospital settings [13]. Approximately 70% of participants in our study used them at admission. One possible reason for the decreased use of absorbent products in the intervention group may be an effective goal‐setting process facilitated by the SBAR technique. For example, nurses and occupational therapists shared a patient’s incontinence issues (situation), and assessed the reason for absorbent product use (background). If the reason was the burden of nursing staff due to the patient’s physical and cognitive abilities, not because of urologic diseases or conditions (assessment), the occupational therapists proposed strategies to enable the patient to use the toilet with minimal assistance. Finally, the occupational therapists shared strategies such as mobility training, environmental modifications, and optimized assistance techniques to facilitate toilet use with nurses (recommendation). Our previous study supported this framework, showing that effective communication fosters a unified approach between professions [19]. Thus, it is reasonable to assume that the SBAR‐based meeting could increase toilet use and reduce absorbent product use.

The use of physical restraints significantly decreased in both groups, reflecting the effectiveness of care provided in the community‐based care ward. Upon initial admission, older patients often experience confusion, making it difficult for healthcare workers to predict their behavior and prompting the use of physical restraints as a precautionary measure against falls [29, 30]. Indeed, approximately half of the patients in this study were subjected to physical restraints upon admission for fall prevention. However, by the time of discharge, the use of physical restraints had decreased to 20% in both groups. This reduction could be attributed to the ward nurses’ enhanced understanding of patients’ behavioral characteristics, fostered by the ward focused on ADL recovery and early discharge planning. Given that the latest Cochrane review concluded that the impact of organizational care on reducing physical restraint use is based on low‐certainty evidence [8], we suggest that the community‐based care ward could serve as a structured environment to minimize the use of physical restraints.

The improvement in the FIM motor in hospitalized older patients suggests a benefit of the CP‐based intervention. In our previous pilot study, improvements in FIM motor scores were observed in both the control and intervention groups, making it difficult to clearly determine the effect of nurse‐occupational therapist collaboration on ADLs [19]. In contrast, the present study showed a significantly greater improvement in the FIM motor gain in the intervention group compared to the historical control group. This difference may be explained by patients’ characteristics at baseline. Our previous study was conducted in a community‐based care ward in a different hospital, where most patients were admitted from their homes and were independent prior to admission. In contrast, in the present study, most patients in both groups resided in nursing homes with assistance, and approximately one‐third had dementia. Generally, it is known that there is little hope for ADL recovery for older patients diagnosed with dementia or those already dependent on ADLs before admission [31, 32]. Moreover, a Cochrane review reported low‐certainty evidence regarding the effectiveness of rehabilitation or care interventions for older patients with dementia [33]. To address this complexity, we provided an integrated care approach [34], which delivered the advantage of integrating fragmented resources by promoting enhanced communication between nurses and occupational therapists. In this study, occupational therapists assessed patients’ maximum abilities, while nurses provided support with their daily activities in the ward. This complementary approach bridged the two professional disciplines, facilitating the integration of therapy into ADLs. Taken together, by targeting older patients with complex needs, such as cognitive impairment and high dependency, this study provides additional evidence supporting the effectiveness of nurse‐occupational therapist collaboration in improving ADLs.

Additional improvements were also observed in patients’ FIM cognitive‐related scores. These findings are consistent with our previous pilot study, which demonstrated that a nurse‐occupational therapist collaboration resulted in positive behavioral changes in older patients [19]. Interestingly, the present study provided further insight into the importance of sharing not only functional abilities but also patients’ preferences and pre‐hospital meaningful occupations during the meeting. Understanding patients’ backgrounds helps healthcare staff shift from a disease‐centered to a person‐centered approach, fostering better relationships with healthcare providers [35]. Conversely, a lack of knowledge about patients’ backgrounds can lead to a decline in the quality of relationships with healthcare providers [36]. By incorporating information related to patients’ backgrounds into the meeting, this collaborative model enhanced the overall quality of care while building positive relationships among patients, nurses, and occupational therapists.

This study has several limitations. First, the participants were recruited from a single facility, which may limit the generalizability of the findings. Second, insufficient data from historical controls introduced selection bias. Although collecting baseline and follow‐up data in clinical settings is crucial for building clinical evidence, many facilities do not consistently monitor such data [37]. This may require researchers’ support to collect outcome data. Additionally, the difference in study periods between the historical control and intervention groups may have introduced confounding factors, particularly due to the COVID‐19 pandemic, which affected hospital stays and healthcare system strain [38]. Cluster infections occasionally occurred during the intervention period, leading to prolonged hospital stays in the intervention group compared to the historical control group. During the earlier phase of the pandemic, which corresponded to the historical control data collection period, rehabilitation services were sometimes restricted to prevent infection, resulting in a higher number of patients who did not receive rehabilitation. Despite these limitations, the study demonstrates the positive effects of the CP model. Future research should consider a multicenter randomized controlled trial to validate these findings.

## 5. Conclusions

This study demonstrated the positive impact of a CP model between nurses and occupational therapists in improving ADLs and reducing disposable absorbent product use in hospitalized older patients. These findings suggest that this model of CP enhances the quality of geriatric care.

## Ethics Statement

The study was carried out in accordance with the Declaration of Helsinki (as revised in Brazil 2013) and was approved by the ethics committee of Gunma Paz University (PAZ21‐36) and Gunma Paz Hospital (No.33).

## Conflicts of Interest

The authors declare no conflicts of interest.

## Author Contributions

K.K., S.H., and N.T. conceptualized and designed the study. K.K., N.N., R.A., and T.O. prepared and coordinated data analyses and interpretations. K.K. and B.L. analyzed the data. C.S., W.M., and B.L. provided statistical support in addition to the critical feedback of the paper. All authors participated in the critical review and approved the submission of the manuscript.

## Funding

This study is supported by the Japan Society for the Promotion of Science, 10.13039/501100001691, 22K17540.

## Data Availability

The data that support the findings of this study are available from the corresponding author upon reasonable request.
